# Migraine and percutaneous patent foramen ovale closure: a systematic review and meta-analysis

**DOI:** 10.1186/s12872-017-0644-9

**Published:** 2017-07-26

**Authors:** Yu-Jie Shi, Jun Lv, Xing-Ting Han, Guo-Gang Luo

**Affiliations:** 1 Department of Neurology, Xi’an Jiaotong University, Xi’an, China; 2 Department of Neurology, The First Affiliated Hospital of Xi’an Jiaotong University, 277 Yanta West Road, Xi’an, Shaanxi 710061 China; 3 Department of Stomatology, Xi’an Jiaotong University, Xi’an, China

**Keywords:** Migraine, Migraine with aura, Patent foramen ovale, Percutaneous patent foramen ovale closure, Right-to-left shunt

## Abstract

**Background:**

The association between patent foramen ovale (PFO) and migraine with aura (MA) is well established. However, the benefits of PFO closure are less certain in patients with migraine without aura (MwoA).

**Methods:**

We systematically searched Pubmed for pertinent clinical studies published from January 2000 to July 2015. The primary end-point was the elimination or significant improvement of migraine symptoms after PFO closure.

**Results:**

Upon screening an initial list of 315 publications, we identified eight studies that included 546 patients. Overall, our analysis indicated a significant improvement of migraine in 81% of MA cases compared to only 63% of MwoA cases. The summary odds ratio was 2.5 (95% confidence interval 1.09–5.73), and the benefits of PFO closure were significantly greater for patients with MA compared to patients with MwoA (*P* = 0.03).

**Conclusions:**

The presence of aura provides a reference standard for the clinical selection of patients with migraine for PFO closure intervention.

**Electronic supplementary material:**

The online version of this article (doi:10.1186/s12872-017-0644-9) contains supplementary material, which is available to authorized users.

## Background

Migraine affects 10–13% of the general population [1] and was ranked seventh in the 2010 Global Burden of Disease study [2]. In 36% of migraineurs, the migraine attack is preceded by a prodromal visual experience known as an aura [3]; migraine with aura (MA) is recognized as a specific migraine subtype. The cardiac anomaly known as patent foramen ovale (PFO), which is characterized by a hole in the heart that did not close properly after birth, has been implicated in the etiology of migraine attacks. Wilmshurst et al. [4] initially reported that PFO closure ameliorated migraine in divers treated for decompression illness. Furthermore, several retrospective observational studies showed that approximately 80% of patients reported improvement of migraine attacks after PFO closure [5–7].

Recently, a particular association between PFO and MA was reported in the literature [8–11]. Several studies reported a significant decrease in the frequency of migraine attacks following PFO closure in patients with MA, whereas patients with MwoA did not benefit from the treatment [12, 13]. In order to clarify the relevance of the aura in the decision to undertake PFO closure, we conducted a meta-analysis to test for population differences in the response of migraineurs to PFO closure.

## Methods

### Literature search strategy

**Fig. 1:**
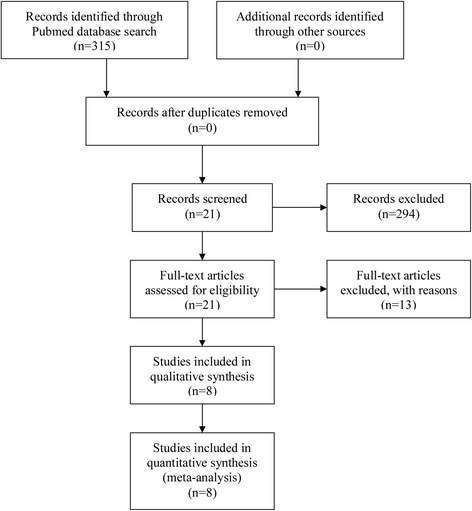
Flow diagram demonstrating the search results. In total, eight reports were included in the meta-analysis

Two investigators attained a consensus on the search strategy and inclusion criteria and independently searched the Pubmed database for relevant articles published between January 2000 and July 2015 using the following search terms: “patent foramen ovale” AND “migraine disorders” OR “migraine” AND “clinical trials” (as topic) AND “humans” (not animals). We only included articles published in English. We also undertook an additional manual search of secondary sources. The Preferred Reporting Items for Systematic Reviews and Meta-Analyses (PRISMA) checklist was followed in the current study (Fig. 1). Moreover, a list of Additional file 1 for the PRISMA flow diagram is shown in Table 1.

**Table 1:** Characteristics of the included studies

Author, year	Length of follow-up	Method of outcome assessment	Grade of evidence	Subgroup	Preclosure	Postprocedural therapy
Schwerzman et al., 2004 [25]	1 Y	A detailed questionnaire	Low	MA	Attack frequency: 1.2 ± 0.8/M Number of large shunt: 27	Aspirin 100 mg/d 6 M Clopidogrel 75 mg/d 1 M
				MwoA	Attack frequency: 1.2 ± 0.7/M Number of large shunt: 6	Ditto
Azarbal et al., 2005 [21]	1 Y	MIDAS questionnaire	Low	MA	NA	Aspirin 325 mg/d 3 M clopidogrel 75 mg/d 3 M
				MwoA	Ditto	Ditto
Slavin et al., 2007 [26]	30 ± 16 M	MIDAS Score	Low	MA	MIDAS Score: 48.3 ± 11.8	NA
				MwoA	MIDAS Score: 43.2 ± 11.9	Ditto
Reisman et al., 2005 [24]	1 Y	Migraine questionnaire	Low	MA	NA	Aspirin 325 mg/d 6 M Clopidogrel 75 mg/d 3 M
				MwoA	Ditto	Ditto
Jesurum et al., 2008 [23]	1.5 Y	Migraine questionnaire	Low	MA	Attack frequency: 5.1 ± 7.5/M Number of large shunt: 51	Aspirin 325 mg/d 6 M Clopidogrel 75 mg/d 3 M
				MwoA	Attack frequency: 4.8 ± 6.7/M Number of large shunt: 20	Ditto
Dubiel et al., 2007 [22]	Mean 38 M	A structured questionnaire	Low	MA	NA	Aspirin 100 mg/d 6 M
				MwoA	Ditto	Ditto
Whal et al., 2010 [27]	5.0 ± 1.9 Y	A structured questionnaire	Low	MA	Attack frequency: 1/d 3 Number of large shunt: 76	Acetylsalicylic 100 mg/d 6 M Clopidogrel 75 mg/d 6 M
				MwoA	Attack frequency: 1/d 1 Number of large shunt: 46	Ditto
Rigatelli et al., 2012 [18]	24–76 M	MIDAS	Low	MA	Attack frequency: 1.1 ± 0.2/M	NA
				MwoA	Attack frequency: 4.2 ± 0.8/M	Ditto

*M* months, *MA* migraine with aura, *MIDAS* Migraine Disability Assessment Test, *MwoA* migraine without aura, *Y* years

### Study selection criteria

Citation abstracts were first screened by two independent reviewers, and complete manuscripts were retrieved if deemed potentially pertinent. The two reviewers independently appraised the identified articles according to the above-mentioned selection criteria, with consensus resolution in cases of disagreement. The inclusion criteria were as follows: (i) observational studies that examined the effect of PFO closure on migraine; (ii) distinction of MA and MwoA as defined by the criteria of the International Headache Society; (iii) PFO detected either by transthoracic echocardiography with peripheral injection (cTTE), transoesophageal echocardiography with peripheral injection (cTEE), or transcranial Doppler ultrasonography with injection (cTCD); (iv) participants were 18 to 60 years of age; (v) a minimum of 10 patients in each group; and (iv) mean duration of follow-up of at least 6 months. The main exclusion criteria were as follows: (i) headache plausibly caused by conditions other than PFO, and (ii) conference abstracts or published data uninformative about patient outcomes. For outcome scoring, the primary efficacy end-point was the cure of migraine or at least 50% improvement in the severity of migraine symptoms. The secondary efficacy end-point represented any difference between basal and final scores in tests including the Migraine Disability Assessment Test (MIDAS) or the Headache Impact Test-6 (HIT-6).

### Study quality assessment and data extraction

The two independent reviewers judged the quality of each included study using the Grades of Recommendation Assessment Development and Evaluation (GRADE) assessment system [14]. The two independent reviewers prepared a formal and un-blinded abstract of each study on pre-specified forms, and resolved any important differences of opinion by consensus agreement.

### Statistical methods

We selected the odds ratio (OR) as the parameter for summarizing each study. Heterogeneity was evaluated using the X^2^ test and I^2^ statistics. Briefly, fixed-effect methods were used when *P* > 0.1 and I^2^ ≤ 50%. When *P* < 0.1 and I^2^ > 50%, we first identified the origin of heterogeneity, and then carried out subgroup analysis focusing on the attribution of particular factors causing the inconsistency. If there were statistical inconsistencies in the absence of clinical inconsistency, we used a random-effects model. Finally, we calculated the summary ORs and 95% confidence intervals (CIs). In addition, we prepared a funnel plot to depict the possibility of publication bias. We reported two-tailed *P* values throughout, using a 0.05 threshold for hypothesis testing, when applicable.

## Results

### Search results

The search strategy initially yielded 315 articles. After screening the titles and abstracts, 21 articles were further investigated [6, 14–32], of which, eight different articles comprising 546 patients were thoroughly reviewed [18, 21–27].

### Definitions

Most participants had been referred for secondary preventive surgery after presumed paradoxical embolism attributed to PFO. However, the participants included in Rigatelli et al. [18] and Azarbal et al. [21] had no previous history of cryptogenic stroke or transient ischemic attack. PFO was evaluated by cTEE in four of the selected studies [22, 25–27], by cTCD in one [21], and by cTCD or cTEE in the remaining three studies [30, 33, 34]. In three studies [18, 21, 26], the frequency and severity of migraine were assessed by the MIDAS questionnaire and score. In the remaining studies [22–25, 27], the authors created their own questionnaires to record the severity and incidence of headache.

### Patients and study characteristics

The mean age of the included patients ranged from 39 ± 6 years to 53 ± 11 years. Sixty-nine percent of the participants suffered from MA, while the remainder had MwoA. Seven of the eight studies were retrospective, while the remaining study [18] was prospective. The detailed demographic characteristics of the included studies are reported in Table 1.

### Quantitative synthesis

The effect of PFO closure on migraine was studied in 546 patients (379 MA and 167 MwoA). At follow-up, migraine improved following PFO closure in 306 (81%) patients with MA and in 105 (63%) patients with MwoA. The estimated effect of PFO closure was reflected by a summary OR of 2.5 [95% CI, 1.09–5.73]. In accordance with the evident statistical heterogeneity [I^2^ = 67%, *P* = 0.003], we carried out subgroup analysis after excluding three ambiguous studies because of their measuring methods, occurrence of different conditions at baseline, inconsistency in the reported length of treatment, and other factors [21, 22, 26]. In the absence of clinical inconsistency, we used a random-effects meta-analytical approach to combine the results of the individual studies. The overall difference in therapeutic efficacy between the MA and MwoA groups was statistically significant [Z = 2.16, *P* = 0.03].

### Assessment of publication bias

**Fig. 2:**
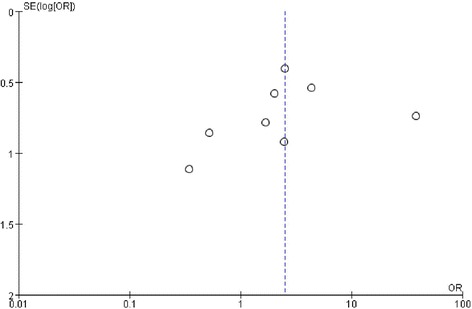
Funnel plot of migraine improvement among patients with migraine after treatment with percutaneous patent foramen ovale (PFO) closure. The asymmetry suggests some publication bias favoring small effects

Figure 2 depicts a funnel plot for the eight studies of percutaneous PFO closure used to treat migraine. The funnel plot demonstrates asymmetry, which suggests possible publication bias.

## Discussion

**Fig. 3:**
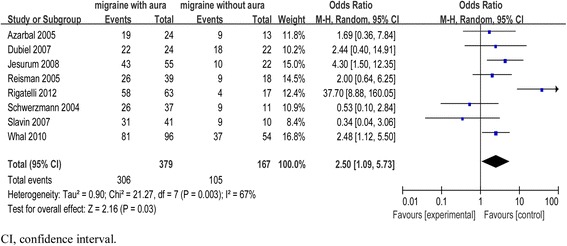
Forest plot of migraine improvement among patients treated with percutaneous patent foramen ovale (PFO) closure

As depicted in Fig. 3, the association between PFO closure and headache improvement was stronger in patients with MA compared to patients with MwoA. It has been postulated that PFO promotes migraine because of incomplete transit of venous blood through the pulmonary circulation. As a consequence, serotonin and microembolic signaling factors, which are normally metabolized in the pulmonary circulation, enter the cerebral vasculature. Upon attaining a threshold concentration in the arterial circulation, certain vasoactive substances provoke attacks of cortical spreading depression (CSD), thus precipitating the aura [13, 33, 35]. Alternately, it is possible that the long-term shunting of vasoactive agents may reduce the threshold for spontaneous migraine initiation [34].

We propose that the reduced concentrations of serotonin and microembolic signaling agents on the arterial side after successful PFO closure ameliorate migraine attacks mediated by CSD. This explanation seems particularly fit to account for the greater reduction in MA attacks after PFO closure. However, it is less clear how MwoA could be related to left-right shunt, given the weaker association between the migraine attacks and PFO closure.

The results of this meta-analysis are encouraging with respect to the benefits of PFO closure, indicating the disappearance or significant improvement of the incidence or severity of MA attacks after surgery, mainly based on retrospective reports. These general findings stand in contrast to results of the Migraine Intervention With STARFlex Technology (MIST) trial [9], which included patients with frequent, disabling, and drug-resistant MAs. This discrepancy could be explained in two ways. First, the length of follow-up in the MIST trial was only 3–6 months, and the early benefits of PFO closure may have been moderated by a possible transient adverse reaction to device implantation. Second, in the MIST trial, a single device type, which varied in size, was implanted regardless of the specific interatrial septum characteristics among the patients. However, other studies included in the present meta-analysis took into consideration the advantages of different devices.

### Limitations

Several limitations in the present meta-analysis are worth mentioning when drawing conclusions regarding the benefits of PFO in MA patients. First, most of the included studies were retrospective, suggesting that a recall bias cannot be excluded. Second, the post-surgical therapy and protocol for assessing the outcomes differed among studies. Third, as noted above, the surgical procedures employed several different devices. Finally, based on the contact with the corresponding authors, the baseline data on sex and age were not recorded in three of the included studies [21, 22, 26].

## Conclusions

Our systematic literature review and meta-analysis confirmed that the presence of aura serves as a predictor for obvious improvement of migraine headache symptoms after PFO closure. Thus, the presence of aura provides a reference standard for the clinical selection of patients for PFO closure surgery. Due to the possibility of bias arising from this retrospective analysis, we perceive the future need for prospective controlled randomized trials to demonstrate conclusively the prognostic value of aura for patient outcomes after PFO closure.

## Additional file

Additional file 1: PRISMA 2009 Flow Diagram (DOC 62 kb)

## Abbreviations

CSD: cortical spreading depression
cTCD: transcranial Doppler ultrasonography with injection
cTEE: transoesophageal echocardiography with peripheral injection
cTTE: transthoracic echocardiography with peripheral injection
GRADE: Grades of Assessment Development and Evaluation
HIT-6: Headache Impact Test-6
MA: migraine with aura
MIDAS: Migraine Disability Assessment Test
MwoA: migraine without aura
PFO: patent foramen ovale
