# Supplementary material for: Migraine and percutaneous patent foramen ovale closure: a systematic review and meta-analysis
Source: BMC Cardiovasc Disord. 2017 Jul 26;17:203. doi: 10.1186/s12872-017-0644-9 (PMC5530487; doi:10.1186/s12872-017-0644-9)
Supplement: Additional file 1: — PRISMA 2009 Flow Diagram (DOC 62 kb) [file 12872_2017_644_MOESM1_ESM.doc]

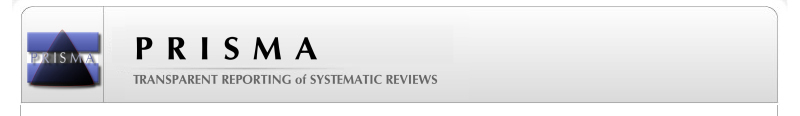
**PRISMA 2009 Flow Diagram**

**Screening**

**Included**

**Eligibility**

**Identification**

Records identified through database searching
(n=315)

Additional records identified through other sources
(n=0)

Records after duplicates removed
(n=0)

Records screened
(n=21)

Records excluded
(n=294)

Full-text articles assessed for eligibility
(n=21)

Full-text articles excluded, with reasons
(n=13)

Studies included in qualitative synthesis
(n=8)

Studies included in quantitative synthesis (meta-analysis)
(n=8)
